# Patients' understanding of genetic susceptibility testing in mainstream medicine: qualitative study on thrombophilia

**DOI:** 10.1186/1472-6963-7-82

**Published:** 2007-06-12

**Authors:** Paula M Saukko, Sian Ellard, Suzanne H Richards, Maggie H Shepherd, John L Campbell

**Affiliations:** 1 ESRC-Centre for Genomics in Society, University of Exeter, UK; 2 Royal Devon & Exeter NHS Foundation Trust & Peninsula Medical School, Universities of Exeter and Plymouth, Exeter, UK; 3 Primary Care Research Group, Peninsula Medical School, Universities of Exeter and Plymouth, Exeter, UK; 4 Institute of Health Service Research, Peninsula Medical School, Universities of Exeter and Plymouth, Exeter, UK

## Abstract

**Background:**

UK and US policy initiatives have suggested that, in the future, patients and clinicians in mainstream medicine could use genetic information to prevent common illnesses. There are no studies on patients' experience and understanding of the process of testing for common genetic susceptibilities in mainstream medicine.

**Methods:**

Qualitative interviews with 42 individuals who had undergone testing for a genetic susceptibility for deep vein thrombosis in primary and secondary care in the UK.

**Results:**

Some participants, often from higher social classes, had a good understanding of the test and its implications. They had often sought additional information on thrombophilia from relatives and from the Internet. Others, often from less privileged backgrounds, had a poorer understanding of the test – seven individuals were unaware of having had the genetic test. Features of genetic information led to misunderstandings: (i) at referral, (ii) when communicating results, and (iii) when making sense of the implications of testing. Participants' accounts indicated that non-specialist doctors may feel obliged to refer a patient for a genetic test they know little about, because a patient requests it after a relative had tested positive. Sometimes a referral for a genetic test was lost under information overload when multiple tests and issues were considered. The inconsistent and informal ways of communicating test results – for example by phone – in mainstream medicine also led to confusion. Participants did not generally overestimate their risk, but some were uncertain about whether they were taking the right preventive actions and/or whether their children were at risk. Information about genetic susceptibilities was difficult to make sense of, as it related to ambiguous risks for participants and family members, complicated and unfamiliar terminology and multiple genes and preventive strategies.

**Conclusion:**

Policy visions of clinicians and patients in mainstream medicine seeking and using genetic information at their own initiative may not be realistic. Patients need more direct support in making sense of genetic information, if this information is to bring the anticipated health benefits, and not fuel health inequalities or create ethical problems. Clinicians in secondary and primary care need guidance to help them introduce genetic tests, communicate their results and explain their implications.

## Background

### Transformation of genetics

In the early and mid 20^th ^century genetics and genetic counselling were expected to provide family planning or contraceptive/sterilization advice to couples in order to avoid passing on "bad genes" [1]. Later these eugenistic ideas became suspect. The role of genetic counselling changed into provision of "non-directive" advice on the likelihood of inherited disease or disability [2].

As long as genetics was associated with mostly incurable single-gene disorders it appeared as an ethically contentious matter best managed by clinical genetics. The development of preventive strategies for conditions such as familial hypercholesterolaemia has changed the nature of genetics. The UK Government's White Paper on Genetics has suggested that as the science advances, testing for genetic susceptibilities could, in the future, become a routine part of primary prevention of common diseases, such as heart disease, diabetes and cancer [3,4]. There are currently very few valid genetic tests for susceptibility to common illnesses, but the US Centers for Disease Control (CDC) is developing an online tool (Family Healthware) that would enable people to collect information about family history of common illness and personal behaviour and to receive preventive advice online and/or from their physician [5]. UK policy makers are developing similar initiatives [6].

The initiatives to incorporate genetic information into everyday healthcare and into online self-health tools reflect developments in science and contemporary health care policies. New policies envision individuals to become "experts" in preventing disease and "choosing" health and healthcare [7,8]. Visions of individuals taking charge of their health promise to democratise medicine but also shift responsibility for health from professional services towards the individual [9,10].

Qualitative studies on people's views on being experts in relation to their health have discovered that some people feel that "knowing" is the "doctor's job" [11] or resent the idea of "choosing" best health care, noting it should be "offered automatically" [12]. Quantitative studies indicate that individuals with less education or lower incomes or from ethnic minorities are least likely to seek health information at their own initiative, more likely to place their trust in doctors and less likely to take preventive actions [13,14]. Similar inequalities have been observed in breast cancer genetics; ethnic minorities and people with a lower socio-economic status are significantly less likely to seek risk assessment and testing [15].

In short, the ethos in genetics-related health policy is shifting from protecting patients from potentially harmful information towards enhancing public health through encouraging clinicians and patients to seek and use information about genetic susceptibilities to prevent disease [16]. In this article we explore how the new ethos works in practice by examining how patients experience and understand testing for a genetic susceptibility in routine clinical practice.

### Experiences of testing

Psychological studies have indicated that genetic testing for single-gene diseases does not generally increase individuals' anxiety [17,18]. It has been suggested that individuals who seek genetic testing are already concerned about their susceptibility to disease, and testing may help them to cope with stress and uncertainty [19].

With the increasing emphasis on prevention, research has focused on behavioural effects. Genetic testing has not been observed to motivate smoking cessation in a community setting [20] and its effects on cancer screening behaviour has been contradictory [21]. But a clinic-based study reported haemochromatosis testing to motivate venesection, a simple preventive strategy [22].

Many studies have reported shortcomings in lay understanding of genetic information. Qualitative studies have observed that individuals have difficulties in understanding, or accepting, inheritance patterns [23]. People also adopt various lay models of inheritance, thinking that they are more likely to inherit a genetic susceptibility from family members, who are "like" them in terms of physique or personality [24].

Quantitative studies have observed that, for example, patients with familial hypercholesterolaemia (FH) have a poor understanding of risk and inheritance [25]. Yet, in a FH trial all participants correctly reported their genetic test results [26]. It has been reported that genetic counselling enhances understanding of breast cancer genetic knowledge, particularly among the better educated [27]. In a study investigating the use of genetic tests results in motivating smoking cessation in the community, 45% of low-income African-Americans did not understand their test results [20]. A research project on patients' experiences of testing for predisposition to haemochromatosis (iron overload) in a clinic-setting found that a third of participants did not know how many mutations they had inherited [22], and healthy individuals tested for haemochromatosis were observed to be nonchalant about the condition in a qualitative study [28]. In the only study conducted so far on patients' perceptions of genetic testing for thrombophilia in primary and secondary care, 79% of the participants could not correctly approximate their risk of deep vein thrombosis [29].

Overall, the literature suggests that genetic testing does not necessarily cause psychological anxiety, but neither does it necessarily motivate behaviour change. Literature also indicates that patients' understanding of genetic testing and its implications is often patchy, particularly in community settings. This partly reflects the fact that non-specialist doctors in primary and secondary care do not have a good knowledge of genetics and do not often perceive it as pertinent to their work [30-35].

Recently, guidelines and tools have been produced for communicating about genetic information [36] and for evaluating the quality of information provided [37]. Programmes for training specialist general practitioners and nurses to support the integration of genetics into primary care have also been developed [3]. Still, the processes that guide patients' experiences and understanding of genetic information in mainstream medicine have not been examined. This article explores these processes.

### Thrombophilia

We chose to focus on patients' experiences of genetic testing for thrombophilia, because it offers a good model for understanding genetic susceptibility testing. Thrombophilia is: (i) common, (ii) associated with low risk, (iii) can be tested in mainstream medicine, and (iv) people tested can be offered advice on preventive strategies. The most common allele associated with the susceptibility, factor V Leiden (FVL), is present in 1:25 of Caucasians, and being heterozygous (having inherited the allele from one parent) for FVL is associated with 0.6% annual risk of deep vein thrombosis among healthy individuals [38]. Other rarer thrombophilias, such as Antithrombin deficiency, are associated with more significant risk [39]. Thrombophilia is also associated with miscarriage [40]. Preventive strategies include: avoidance of the combined oral contraceptive and hormone replacement therapy (HRT); precautions/prophylaxis to be adopted in high risk situations (surgery, pregnancy and postpartum, long flights); and avoidance of smoking and weight. Thrombophilia can be tested for in primary and secondary care, without the necessary involvement of clinical genetics [41,42]. The clinical utility of thrombophilia testing has been a matter of debate [43]. A recent UK Health Technology Assessment report did not recommend thrombophilia screening for all women before going on the oral contraceptive pill, but testing is recommended for individuals with a personal or family history of deep vein thrombosis, or a relative with thrombophilia [39]. Although thrombophilia testing receives much less public attention than breast cancer genetics, it is one of the most common genetic tests in the US and the UK [29,44].

## Methods

We first identified typical situations in which individuals are referred for factor V Leiden testing, by analysing laboratory requests received by the Royal Devon & Exeter NHS Foundation Trust Molecular Genetics Laboratory between April 2002 and March 2004. We used descriptive statistics to identify patterns of referral in terms of: (i) source of referral; (ii) gender of patient; (iii) reason for; and (iv) test results.

The descriptive analysis informed the maximum variation sampling for a qualitative interview study. We invited a total of 97 participants for semi-structured interviews via their referring doctors (general practitioners or consultant haematologists). We invited participants from both primary and secondary care, with different reasons for referral, women and men, with different test results and from socioeconomically diverse areas. We chose qualitative methods, because they allow for exploring processes that lead to quantitative outcomes, such as patients' understanding, in the new area of genetic susceptibility testing in mainstream medicine [45].

After obtaining ethical approval we conducted interviews with consenting participants between January and November 2004. Most interviews took place in the participant's home. The interviews followed the format of discovery interviews [46]. We asked the respondents to relate their experience of being tested in a chronological order; how they came to have the test, why they decided to have it, how they learnt the results and reacted to them, what they did with the information afterwards, and what they thought of the process of testing as a whole. The schedule provided a naturally flowing chronological structure for the interviews that covered the process of testing but was open-ended enough to allow the participants to expand to other areas they deemed important. The interviews were recorded and transcribed. They were analysed for themes using the constant comparative method [47], which inductively seeks to identify themes that emerge across cases. The thematic analysis was aided by the NVivo qualitative software. The interviews were all conducted and interpreted by one experienced researcher (PS); a sample of six interviews was read independently for themes and subgroups by two other members of the team (SR, MS) to enhance reliability.

## Results

### Typical referral situations

Of the 390 referrals (307, 79% women), 190 (49%) originated from primary and 173 (44%) from secondary care, 27 (7%) originating from outside Devon. The majority (295, 76%) of individuals tested had normal results, whilst 94 (24%) were heterozygous and one (0.3%) was homozygous for FVL. The most common reasons for referral were: (i) personal history of deep vein thrombosis (DVT) (115/390, 30%); (ii) family history of DVTs (74/390, 19%); (iii) family history of genetic thrombophilia (67/390, 17%); and (iv) recurrent miscarriages (45/390, 11%).

The referral patterns indicated that thrombophilia testing was adopted by both primary and secondary care clinicians, and encompassed both individuals who have had a DVT, and healthy individuals who have a family history of thrombophilia or DVTs.

### Qualitative themes

Of the 97 individuals invited, 42 consented to interview. Twenty-five of the 42 participants reported having been referred to the test because of family history of thrombophilia or DVTs, ten were referred because of personal history of DVTs, 5 were referred for other reasons (such as miscarriages) and seven were unaware of being tested. Most of the participants (37/42) were women; 22 belonged to social class I and II, whilst 20 belonged to social class III, IV and V [48]. Twenty-six individuals with positive results for a thrombophilia marker (participants had often been screened for a panel of five markers) responded to our invitation to participate, but we only recruited nine participants who had normal results (seven were unaware of having had the test). The slanting of the sample towards individuals with thrombophilia should be borne in mind when interpreting the findings.

One theme that emerged from the interviews was that participants did not consider thrombophilia testing special even if it identified a genetic susceptibility; we have discussed this theme elsewhere [49]. Another prominent theme that emerged was that participants' understanding of the test, its results and implications varied widely. This article focuses on this diversity in understandings of the test, exploring processes that lead to better or worse understanding.

The understanding of the individuals did not fall into clear categories but lay on a spectrum. Some participants understood the test well; they knew they had had the test, recalled its results (including the marker they had inherited) and understood the familial and preventive consequences. Others had a fair or poor understanding of the test. Some presumed their test results were normal, even though they could not recall receiving their results. Some knew they were positive for thrombophilia but did not know which marker they had inherited even though different markers are associated with differing risks; they were also unsure about whether or not they were doing the right things to prevent DVTs. Seven participants were unaware of having had the genetic test.

The participants who understood the test well derived predominantly from social class I and II, and participants with poorer understanding were predominantly from social class III, IV and V.

Three sub themes emerged from the interviews that brought into relief the processes that led to differences in understanding: (i) the referral situation, (ii) receiving and interpreting results, and (iii) understanding of the risk and the implications of testing.

### The referral situation

Individuals, who understood the test well, sometimes described the information provided by the doctors as "comprehensive" (Participant 034); other times they commented that for their general practitioner "it was actually the first time she had heard" of factor V Leiden (025). Well-informed participants, who were referred for the test based on family history of thrombophilia had often acquired a basic understanding of thrombophilia prior to testing from knowledgeable family members. When relatives had provided individuals with reasonably good information, which was complemented by the doctor's advice, their understanding of the test at referral was straightforward, as illustrated by the account of a young, well-educated woman:

*Mum just said that there was a test that me and [my brother] had to have to see, if we'd got this factor V Leiden. ... And when I went in with Dr H he just told me what it was*. [09]

Sometimes the information provided by relatives had been less clear, but participants had sought further information from the Internet prior to contacting a clinician so that they "probably knew all that [the doctor] told and more already" (021). Occasionally the participant had taken on the collection of information on behalf of the entire family, particularly in cases where the participant deemed other family members to have difficulties in accessing and understanding information. A community care worker had consulted the Internet on behalf of her family after her brother tested positive for factor V Leiden when diagnosed with a DVT, from complications of which he later died. She also noted that such information seeking was necessary in the case of thrombophilia but not in relation to well-understood and supported illnesses, such as diabetes:

*We didn't know anything about it, so we sort of just looked [on the Internet], and basically we did it for everybody else, you know. Obviously, my mum and dad was concerned because of [my brother]. ... But no, I don't think [we usually consult the Internet]...even with Andrew's [partner's pseudonym] diabetes we've never gone on the Net for that. But that's totally different. A diabetic nurse is so good. We've had loads and loads of information for that*. [025]

Less informed participants had also often been referred for testing on account of a family history of thrombophilia or DVTs, but they frequently indicated that their relatives had not provided them with much background knowledge. They were more likely than well informed participants to report that their doctor had not provided them with good information, saying, for example, that he "never explained [the test] properly" (030). Others acknowledged they had trouble understanding information, stating that it "went over our head" (023). A middle-aged male accountant had asked for the test, after his brother's doctor recommended all family members to be tested following his DVT; the brother had not told him much about the condition, and the participant ended up knowing he was positive but did not know the marker. He considered that he was provided with little information, as the doctor presumed he knew about the test as he requested it:

*She [did not explain the test] in so many words because of the fact that I'd asked for a test myself ... I think that she assumed that I knew what I was being tested for. And as far as I am aware it was just a blood test that had to be done and we went from there *[018]

Two of the participants who were unaware of having had the genetic test had most likely been referred to it in a consultation over prescribing the oral contraceptive or HRT. There had been a difference in opinion between the clinician and the patient, and the test was probably used to mediate the difference and was lost under the main issue of prescribing. Five participants who were unaware of having had the genetic test had had several DVTs or cerebrovascular events. They related that they sometimes had to "insist" on proper medical attention (for example, when experiencing an acute DVT) (026). Yet, they tended to trust their doctors the details of their complex care, such as multiple diagnostic tests, as indicated by the account of a retired pub-keeper:

*I don't remember ever having had the test. ... But I have had lots of blood tests. But I've never known ... really what they were doing with it*. [015]

### Receiving and interpreting results

Participants had learnt of their genetic test results in inconsistent and sometimes informal ways: during a consultation, in writing, on the phone or they did not recall receiving the results. Less informed participants were sometimes confused about the manner in which results would be communicated to them. In several cases they had expected their doctor to call them in person, if the results were positive. In a couple of cases participants thought their results were normal or that they were "clear" because the doctor had not contacted them, only to find out later, when they queried about it, that they "had it" (023). A couple of participants did not at the time of the interview recall receiving the results, presuming they were "fine," as the doctor had not called them (011). One participant was referred to the test after recurrent miscarriages and was given "a negative result when it was positive" by the receptionist (038). Afterwards she had another miscarriage and a daughter who died soon after birth. She admitted that the cause of the tragedy was uncertain, but that in her "heart" she believed it all had to do with clotting, and that if the test results had been conveyed correctly she may have "looked at it a bit closer" during the subsequent pregnancies (038).

On many occasions the more and less informed participants had received the test results in identical manner but reacted to the information differently. After learning their results the well informed participants often sought additional information or advice from their doctors and elsewhere. This is illustrated by a university lecturer's description of her initial reaction to learning on the phone she had inherited factor V Leiden and having been reassured by her doctor not to worry:

*I think I rang up for [the results] ... So, I immediately made an appointment to talk to my doctor about it. ... She looked at me and she said, "You're not obese and you're not a smoker. You don't need to worry". And I felt, "Well, don't just dismiss me" ... [One needs] advice and clarification about what things you do need to worry about and what things you don't need to worry about. I think I've sort of gleaned information by looking at the Internet or talking to my brother or talking to my father. It just makes me much more secure, really*. [010]

A less informed participant, a young woman working in the supermarket, was also communicated the test results over the phone. Yet, rather than pursue further information she concluded that the matter was not important as her doctor had reassured her that she needed to take no specific actions consequent on the test results:

*Eventually someone told me that apparently they weren't supposed to tell me like that. I was supposed to go in and see them, but they had just told me [the results] over the phone. But, mmm, I still didn't go and see the doctor. They didn't call up for me or nothing ... and my GP had said that I would just carry on as normal anyway*. [027]

Many participants, involving both those with good and those with poor understanding, stated that they would have appreciated written information on thrombophilia and prevention, which they could reread at home. They commented that they felt they were not able to "take it all in" (036) during a consultation.

### Understandings of risk and the implications of testing

In the first few interviews patients described themselves as "not too anxious" (037) or "blasé" (039) about thrombophilia. In later interviews we explored this theme systematically by asking the participants whether they considered the risk associated with thrombophilia to be small, medium, or high (this question was not posed when individuals were unaware of having had the genetic test). Of the twenty-three interviewees asked this question only three thought the risk was high, eleven estimated it as small and nine as medium. The three participants who considered their risk high all had a poorer general understanding of thrombophilia.

Most participants did not recall their doctor explaining the risk to them; they had "just figured it out" (017), concluded their risk was medium due to family history ("father had it") (010) or gleaned the understanding from "the way the doctor explained it" (018). Some participants remembered the doctor relating a numerical risk figure, such as "1 in 100" (020); one participant recalled the doctor estimating that the risk was "a lot bigger," but she did not agree with him, as she had been "healthy" (039).

We have reported elsewhere that participants, who knew they had thrombophilia, had stopped taking the combined contraceptive pill and hormone replacement therapy but had not changed their lifestyle [49]. There were no dramatic differences in terms of preventive actions reportedly taken following communication of test results between the well and the less well-informed participants, excluding those who were unaware of being tested.

However, the participants differed in respect of the confidence in their acquired knowledge. A female scientist, who had experienced a DVT in her early thirties after going on the pill and had been identified to have inherited factor V Leiden, had requested a referral to a consultant to ask about prevention. Her knowledge was very detailed and covered all eventualities:

*I often have to travel by plane to Rome. [Professor A] told me that I can, if I want to, have an injection of heparin. He said, if I feel more comfortable, I can just take half of an Aspirin. ... And obviously he tried to explain to me how I can change my life in order to help my circulation and to help myself keep fit. And then I also asked what of contraception? ... So he said that there are different alternatives. So, then I went to a clinic in Exeter, and I have a coil now, so I'm quite happy with it. Then I asked him about pregnancy as well. What should I tell to my doctors and what should I ask them to take care of? And he just said I should be on heparin, I think in the hospital [03]*.

A homemaker, who had experienced a DVT during pregnancy and been identified to have thrombophilia, was much less certain about prevention. The participant also asked the interviewer for advice on prevention:

*Oh, yes [the doctor did mention hormones] I'm not allowed to go on the Pill. ... I just ... do a lot of walking ... But, apart from that, well, I don't know [what to do to prevent clotting]. I just carry on as day-to-day, and I'm pretty happy with that. ... I suppose you could tell me the most high risk factor of it all, because I don't know a lot about it to be honest*. [037]

Most of those participants who had had DVTs but were unaware of having had the test, said they would want to know, "if it is heritable" to help their children. Some of them stated they had "suspected" (035) that abnormal clotting was a hereditary condition. However, their lack of understanding did not enable them to clarify their suspicions or to inform their children about familial risk:

Yes, [I would like to know if there is a risk in my family] especially, if it might help my offspring ... Because our elder daughter has got very big legs. She's always had very sturdy legs but they ... they could give her problems, I think, later on. [026]

## Discussion

We do not want to argue that genetic information is always "exceptional" or different from other medical information, or that patients necessarily perceive it as such [49]. Some features of genetic information, however, led to misunderstandings in thrombophilia testing in mainstream medicine at referral, when receiving and interpreting results, and when making sense of their risk or the implications of testing.

Based on our findings genetic information can create an unusual situation at referral. A non-specialist doctor may feel obliged to refer a patient for a genetic test they may know little about, simply because a patient requests it after a relative has tested positive. Referral for a genetic test in mainstream medicine may also happen in a complex clinical context, such as when prescribing or in relation to an acute or chronic illness involving many diagnostic tests. In such a situation the genetic test, with its familial and other specificities, may disappear under information overload for the patient and/or the clinician.

Our study also indicates that the inconsistent and informal ways of communicating test results in mainstream medicine may not be suitable for relaying genetic susceptibility test results, which are difficult to convey and have implications for relatives, regardless of whether they are abnormal or normal. Patients' reports also suggest that clinicians may be concerned about making patients anxious about a genetic susceptibility and may reassure them in a manner, which was perceived as uninformative or led to ignorance and confusion.

Most participants did not recall the doctor explaining their risk, but they had gleaned a sense of the risk being low from their family history, which is also reported in other studies[24], or from the doctor's behaviour. Participants, who understood the test well, estimated their risk as small or moderate and were confident about their knowledge of prevention. Participants with poorer understanding occasionally considered their risk to be high; generally they deemed themselves to be at a low or moderate risk but sometimes fluctuated between nonchalance and mild worry, such as between being content with limited knowledge and asking the interviewer questions about prevention. Such uncertainty may be conducive of not undertaking preventive actions consistently. Some participants' accounts suggest that shortcomings in communication and understanding about genetic testing for thrombophilia can create worries in sensitive areas of life, such as risks to children or pregnancy. This can fuel feelings of guilt and resentment in the event of a serious medical event, including miscarriage or stillbirth, occurring to the patient or amongst the wider family.

Recent policy initiatives suggest that, in the future, patients would actively seek information, possibly online, about genetic risk factors with a view of preventing common diseases [5,6]. In the light of our findings these visions do not seem realistic. Participants had difficulties understanding genetic information, because it involved complicated and unfamiliar terminology (such as the names of the markers), implied uncertain risks for themselves and family mebers and was associated with multiple markers and preventive strategies. Participants who had sought information about thrombophilia at their own initiative from the Internet or relatives did have a good understanding of the condition. But many of our participants, particularly those with lower socioeconomic status, did not go out their way to pursue health and risk information at their own initiative; many expected their doctors to take the lead in explaining the testing process, its results and risks and to provide them with direct advice. Similar findings about disadvantaged people's reluctance to seek information have been reported in relation to health behaviour in general [13] and in relation to genetics in particular [15].

The fact that some patients were unaware of having had the thrombophilia test or had a poor or fair understanding of what was being tested for and its results also compromises informed consent. The impact of thrombophilia testing may be much slighter than in testing for Huntington's disease, but the classical principle of guaranteeing patient's consent and informing them of the possible familial, psychological and social implications of genetic testing remains of importance.

## Conclusion

Recent initiatives have sought to enhance non-specialist clinicians' ability to use genetic information by providing communication guidelines [36], tools to evaluate the quality of information provided [37] and by training genetics specialist general practitioners and nurses [3]. These initiatives are steps in the right direction. However, more support for patients and clinicians in mainstream medicine is needed if the forecast health benefits of preventive genetics are to be reached without deepening health inequalities or compromising the ethical integrity of testing. In particular clinicians need guidance and support, suitable for mainstream medicine, to help them introduce genetic susceptibility tests, communicate their results, preferably in person, and to explain the level of risk and the implications of test results, complemented with accessible written information.

## Competing interests

The author(s) declare that they have no competing interests.

## Authors' contributions

The study was designed by PS, JC and SE. The laboratory referrals were collated by SE and analyzed by PS and JC. The qualitative material was collected by PS and interpreted by PS, SR and MS. PS drafted the paper, and all other authors commented on and revised it. PS and JC are guarantors of the paper.

## Pre-publication history

The pre-publication history for this paper can be accessed here:


